# Development of a Real-Time Risk Prediction Model for In-Hospital Cardiac Arrest in Critically Ill Patients Using Deep Learning: Retrospective Study

**DOI:** 10.2196/16349

**Published:** 2020-03-18

**Authors:** Junetae Kim, Yu Rang Park, Jeong Hoon Lee, Jae-Ho Lee, Young-Hak Kim, Jin Won Huh

**Affiliations:** 1 Graduate School of Cancer Science and Policy National Cancer Center Goyang-si Republic of Korea; 2 Cancer Data Center, National Cancer Control Institute National Cancer Center Goyang-si Republic of Korea; 3 Healthcare AI Team, Healthcare Platform Center National Cancer Center Goyang-si Republic of Korea; 4 Department of Biomedical Systems Informatics Yonsei University College of Medicine Seoul Republic of Korea; 5 Department of Biomedical Informatics Asan Medical Center Seoul Republic of Korea; 6 Department of Emergency Medicine Asan Medical Center, University of Ulsan College of Medicine Seoul Republic of Korea; 7 Health Innovation Big Data Center Asan Institute for Life Science, Asan Medical Center Seoul Republic of Korea; 8 Medical Information Office Asan Medical Center Seoul Republic of Korea; 9 Department of Cardiology Asan Medical Center, University of Ulsan College of Medicine Seoul Republic of Korea; 10 Department of Pulmonary and Critical Care Medicine Asan Medical Center, College of Medicine, University of Ulsan Seoul Republic of Korea

**Keywords:** deep learning, cardiac arrest, Weibull distribution, forecasting, intensive care units, gated recurrent unit

## Abstract

**Background:**

Cardiac arrest is the most serious death-related event in intensive care units (ICUs), but it is not easily predicted because of the complex and time-dependent data characteristics of intensive care patients. Given the complexity and time dependence of ICU data, deep learning–based methods are expected to provide a good foundation for developing risk prediction models based on large clinical records.

**Objective:**

This study aimed to implement a deep learning model that estimates the distribution of cardiac arrest risk probability over time based on clinical data and assesses its potential.

**Methods:**

A retrospective study of 759 ICU patients was conducted between January 2013 and July 2015. A character-level gated recurrent unit with a Weibull distribution algorithm was used to develop a real-time prediction model. Fivefold cross-validation testing (training set: 80% and validation set: 20%) determined the consistency of model accuracy. The time-dependent area under the curve (TAUC) was analyzed based on the aggregation of 5 validation sets.

**Results:**

The TAUCs of the implemented model were 0.963, 0.942, 0.917, 0.875, 0.850, 0.842, and 0.761 before cardiac arrest at 1, 8, 16, 24, 32, 40, and 48 hours, respectively. The sensitivity was between 0.846 and 0.909, and specificity was between 0.923 and 0.946. The distribution of risk between the cardiac arrest group and the non–cardiac arrest group was generally different, and the difference rapidly increased as the time left until cardiac arrest reduced.

**Conclusions:**

A deep learning model for forecasting cardiac arrest was implemented and tested by considering the cumulative and fluctuating effects of time-dependent clinical data gathered from a large medical center. This real-time prediction model is expected to improve patient’s care by allowing early intervention in patients at high risk of unexpected cardiac arrests.

## Introduction

### Background

In-hospital cardiac arrest (IHCA) is etiologically different from out-of-hospital cardiac arrest owing to the variety of underlying illnesses in hospitalized patients. Unfortunately, despite efforts to improve survival following IHCA, outcomes have not significantly improved over the last few decades [1,2]. In particular, unexpected cardiac arrest is the most serious adverse event related to death in intensive care units (ICUs). The common reasons for cardiac arrest in critically ill patients are severe respiratory insufficiency and hypotension.

Several studies have reported that mortality after IHCA was associated with the timing of cardiac arrest (day vs night shift), type of institution (teaching vs nonteaching hospital), and subsets of patients (ie, age and sex of patients) [3-5]. However, these are not preventable factors. To reduce mortality, we need to be able to predict which critically ill patients are at high risk for IHCA before arrest and the actionable factor to reduce the risk of cardiac arrest. Although many arrests are preceded by clinical deterioration that is either unrecognized or not managed effectively, the complex data of ICU patients make it difficult to model and monitor early warning signs [6]. An additional problem with arrest prediction is the nature of time to event (TTE); we would like to be able to predict not only whether or not cardiac arrest will occur but also when that event will occur [7].

Given the complexity and time dependency of ICU data, machine learning–based methods including the deep learning–based early warning system and gradient boosting machine have provided a good basis to develop risk prediction models using large clinical data contained within electronic medical records [8-12]. Specifically, several deep neural network algorithms have been applied to develop an early warning system for cardiac arrest to predict IHCA a few hours before the event [12,13]. However, in the time series data, constructing the label of the data by assuming the dependent variable is binary has various risks because the time for the onset of symptoms associated with cardiac arrest varies from patient to patient. In this study, rather than simply predicting the probability of cardiac arrest at the current time by binary classification, parameters of the Weibull distribution were used to predict the distribution of the probability of occurrence over time. This allows us to predict when cardiac arrest will occur at this time point, which will enable clinicians to alter the clinical trajectory to prevent cardiac arrest.

### Objectives

This study aimed to develop a real-time deep learning model to predict the risk of cardiac arrest in critically ill patients in a medical intensive care unit (MICU). Then, we evaluated the performance of this system depending on the remaining time from the event occurrence.

## Methods

### Study Design and Subjects

**Figure 1:**
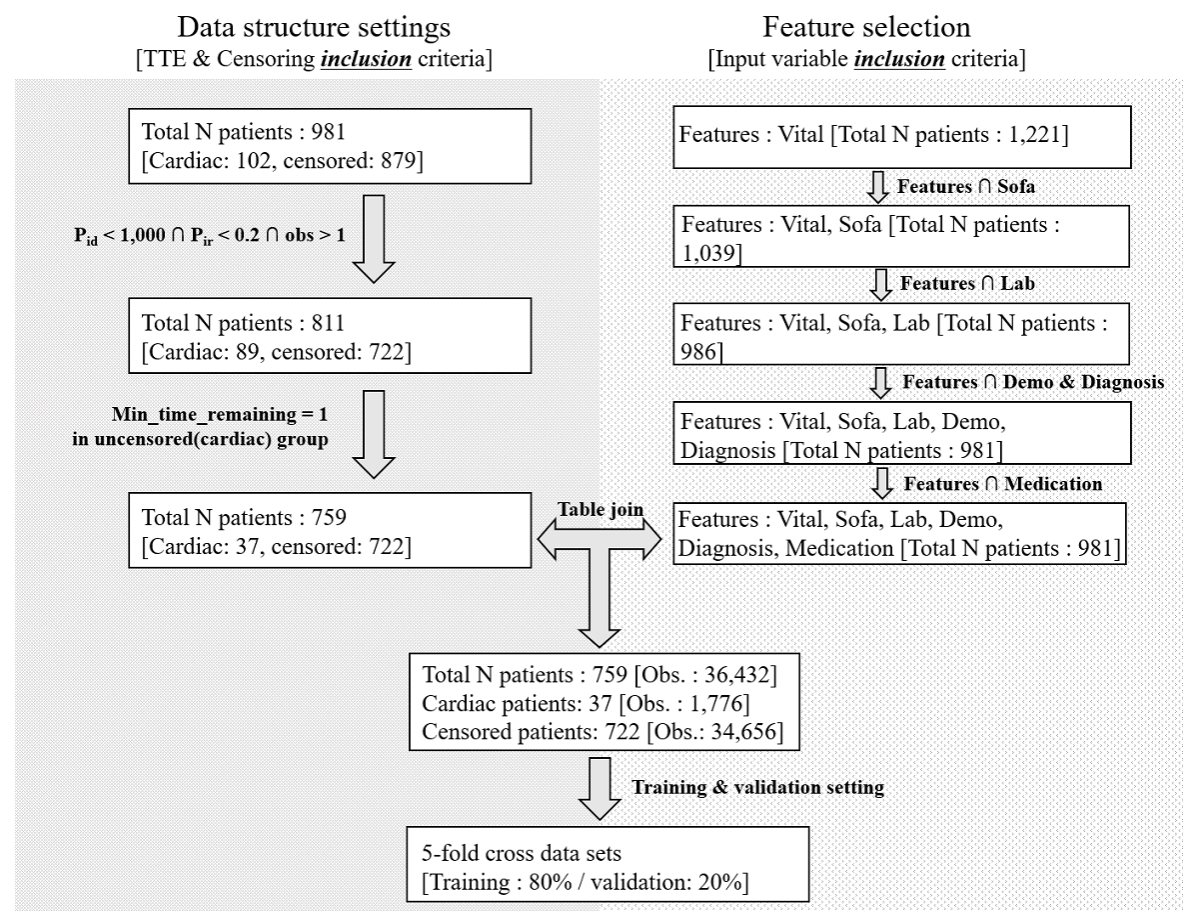
Data preprocessing flowchart. Obs: observation; TTE: time to event.

We conducted a retrospective study with patients admitted to the MICU at the Asan Medical Center in Seoul, South Korea, between January 1, 2013, and July 31, 2015. For the development of a deep learning–based prediction model of cardiac arrest in critical ill patients of the MICU, we identified 759 distinct patients aged 18 years or older who stayed in the MICU for 1 day or more (Figure 1). All clinical data were extracted from our deidentified clinical data warehouse [14]. The extracted clinical data were categorized by patient demographics, diagnosis, medication, vital signs, medication, and inputs and outputs (Multimedia Appendix 1). As most clinical data were time series data, they were automatically recorded using patient monitoring devices. Vital signs and laboratory test data were collected at equally spaced intervals of 1 hour and 1 day, respectively.

**Figure 2:**
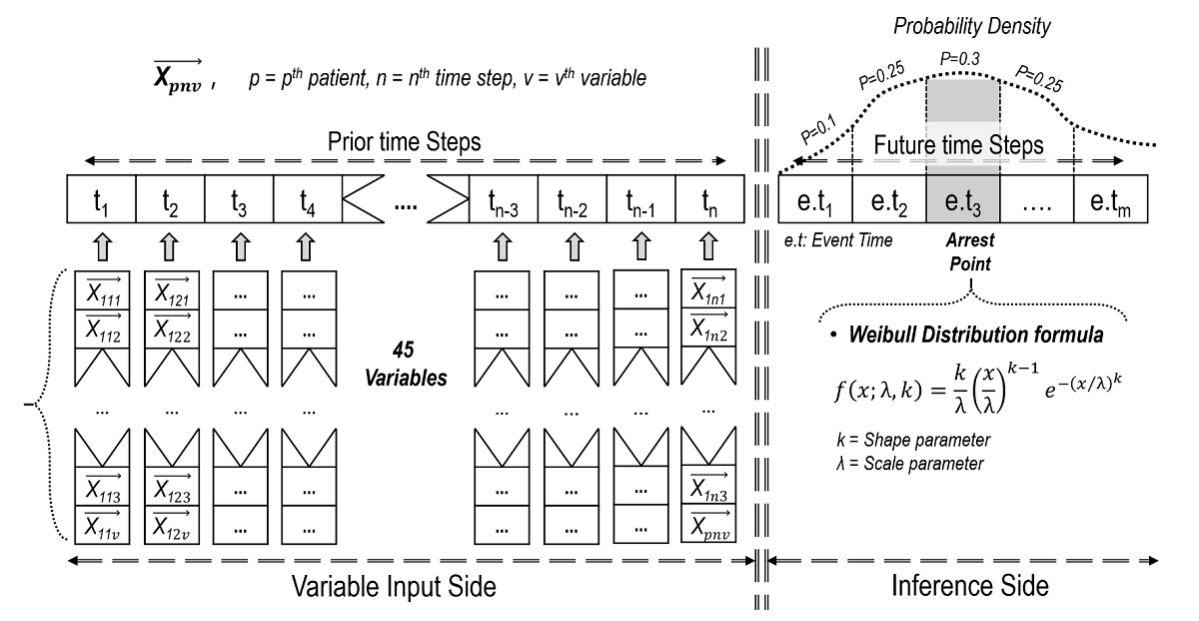
Character-level gated recurrent unit structure combined with the Weibull distribution.

The data were preprocessed in 2 ways. First, we selected features that patients have in common (see Feature selection in Figure 1). The features were divided into 6 broad categories (vitals, Sequential Organ Failure Assessment scores, laboratory results, demographics, diagnosis, and medications), and 45 common variables of 981 patients were selected. Second, we created a data pipeline to fit a gated recurrent unit (GRU) algorithm structure (see Data structure settings in Figure 2). Patient observations that had too many missing values were excluded to prevent biased model estimation. To filter out observations with many missing variables, 2 criteria were used: P_id_ and P_ir_ (Figure 1). P_id_ refers to the amount of missing observations after 2 tables (data pipeline fitted for the GRU structure and features) were joined. The threshold for P_id_ was set to 1000. P_ir_ is the ratio approach and is calculated as P_id_ divided by the total observations of patients in outcome variables. The threshold for P_ir_ was set to 20%. Furthermore, a threshold (τ) is defined to prevent large values of remaining hours in the uncensored group (ie, patients who did not experience cardiac arrest) from causing biased model estimation. For instance, if the uncensored group has many values greater than many hours (ie, 72 hours), then the likelihood of cardiac arrest may be estimated to be lower than that in the censored group. In addition, to accurately predict the occurrence of cardiac arrest, it is important to allow the model to learn the relationship between the time remaining just before cardiac arrest (eg, 1-3 hours) and the variables. Hence, only patients whose data were observed at least 1 hour before cardiac arrest were included in the study (Figure 2). Finally, the 2 tables (data structure that fits the GRU algorithm and features) were joined.

This study was approved by the institutional review board of the Asan Medical Center, Korea (institutional review board number 2015-1015). The need for informed consent was waived by the ethics committee as this study involved routinely collected medical data that were anonymously managed at all stages, including data cleaning and statistical analyses.

### Development of Risk Prediction Model

**Figure 3:**
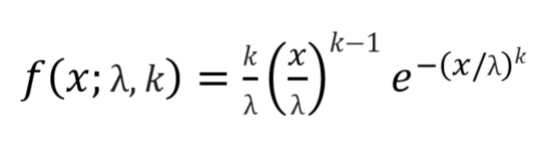
The probability density function of a Weibull random variable. k: shape parameter; λ: scale parameter; x: the quantity of time to failure.

The Weibull distribution, a continuous probability distribution, is a parametric model that can calculate the distribution form of survival time. Given the advantage of parametric models in survival analysis, the Weibull model is often used to estimate failure rate over time [15,16]. The probability density function of a Weibull random variable is shown in Figure 3.

The distribution consists of 2 parameters—the shape parameter *k* and the scale parameter λ. The variable *x* is the quantity of time to failure. Failure rates decrease over time when the shape parameter is less than 1. Conversely, failure rates increase over time when the shape parameter is greater than 1. The scale parameter is a location parameter that affects the width of the distribution. The larger the scale parameter value, the larger the width of the distribution.

*Character-level gated recurrent unit* (Char-GRU) is often used to predict the next token given a sequence of previous tokens [17]. In this research setting, each patient time history (45 historical variables for 1 patient) was preprocessed to become a set of overlapping time histories (see the list of 45 variables in Multimedia Appendix 1). Thus, the data structure consists of a 3-dimensional array: the number of observations × the number of time steps × the number of variables.

Figure 2 represents the structure of the input tensor 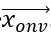 *o, n, v* stands for *o*^th^ observation, *n*^th^ time step, *v*^th^ variable, respectively. For outcome variables, the algorithm estimates 2 parameters of the Weibull distribution by accumulating input variables for up to 48 hours (ie, 2 days). Thus, the 3-dimensional structure of tensor values at the input stage is changed to a 2-dimensional structure through the GRU network, and the parameters of shape and scale are estimated by the last point of the network.

The challenging point of learning the model was related to the censoring feature of the data structure (ie, 1=cardiac arrest occurred or 0=censored). The TTE of cardiac arrest is actually observed data, unlike in the case where the data point is not censored. However, the TTE of cardiac arrest is unknown when the data point is censored. In this study, τ was defined as a threshold value indicating the time to safety in the censored group. We set 72 hours as a threshold based on the median number of hours that patients stayed in the MICU.

### Cost Function and Model Structure

The outcomes of Char-GRU with the Weibull distribution algorithm are 2 parameters corresponding to the shape and scale of the Weibull model. These 2 parameters enable calculation of likelihood through the function proposed in Figure 3. The goal is to obtain the optimal parameter of the Weibull distribution from the sequential patient data; therefore, the negative log of the likelihood is set to the objective function to maximize the likelihood of the training batch. With the objective function, the Char-GRU network parameters were optimized using the Adam stochastic optimization [18].

The total number of patients was 759, consisting of 37 cardiac arrest patients and 722 non–cardiac arrest patients. As 45 variables for 1 patient are repeatedly observed 48 times, the number of observations for cardiac arrest patients is 1776 and that for censored patients is 34,656. Thus, the shape of the input data delivered to the GRU algorithm is a 3-dimensional array of 36,432 × 48 × 45. If 45 variables of a timewise vector are missing, we apply a masking layer that skips the vector and the learning. It is then delivered sequentially to a layer of 50 GRU units. The activation function of this layer is an all hyperbolic tangent function. Next, a fully connected layer of 20 units is connected with the hyperbolic activation function. Finally, the 2 fully connected layers are connected to estimate the shape and scale, the parameters of the Weibull distribution with a softplus activation and exponential function, respectively.

### Cross-Validation Procedure

A fivefold cross-validation test (training set: 80% and validation set: 20%) was implemented to determine the consistency of the model’s accuracy. Overall, 5 models were learned independently from each dataset each time. Time-dependent receiver operating characteristic (ROC) analysis was performed from the aggregated set of the probability of an individual having cardiac arrest in each time step, which was estimated from 5 validation sets [19]. The mechanism for applying the result of the deep learning model to time-dependent ROC analysis is as follows: tensors, which were 3-dimensional in the input level, were passed through the GRU network to estimate the Weibull distribution by learning the shape and scale parameters. The Weibull distribution in this study setting indicates the likelihood (from 0%, low risk, to 100%, high risk) of a heart attack within the next hours from the current point in time. In other words, the time-dependent risk of cardiac arrest for each patient was estimated based on the deep learning model. Thus, the time-dependent risk probability of having a cardiac arrest was passed to the time-dependent ROC analysis.

### Open Source Software

All procedures for data preprocessing and model implementation were conducted through the open source programming languages R and Python. To handle data in the format of a data frame (ie, data table) and an array, 2 open source libraries—Pandas and Numpy—were used. Char-GRU with a Weibull distribution was implemented in Keras (version 2.2.2), a wrapper library from Tensorflow (version 1.10.0), and a representative open source tool supporting the implementation of deep learning algorithms. Detailed concepts and mechanisms at the code level of this algorithm have been well documented in a previous study [16,20]. The ROC analysis was performed based on the R package pROC.

## Results

### Patient Characteristics

A total of 759 patients admitted in the ICU of the Asan Medical Center from March 2015 to March 2017 were enrolled in the study. Descriptive analysis was performed in 2 broad categories: demographics with 3 variables and diagnostic status with 8 variables. The Student *t* test was used for continuous variables such as age; the chi-square test was used for categorical variables such as Diab (ie, 1=diabetes or 0=no diabetes). The results of a descriptive analysis are reported in Table 1. Both age and body weight in the cardiac arrest group were statistically higher than those in the non–cardiac arrest group (ie, censored group; *P*<.001). However, there were no statistical differences between the 2 groups for the remaining variables, including gender and underlying diseases.

**Table 1:** Descriptive statistics of the demographics and underlying diseases of the patients.

Variables			Cardiac group (n=37)		Censored group (n=722)		*P* value (test type)	
**Demographics**								
	Age (years), mean (SD)			62.509 (12.311)		60.526 (13.991)		<.001 (*t* test)
	Weight (kg), mean (SD)			59.734 (13.166)		57.816 (13.435)		<.001 (*t* test)
	**Gender, n**						.**15 (chi-square test)**	
		Male		28		451		
		Female		9		271		
**Diagnosis, n**								
	**Hematologic malignancy**						**.35 (chi-square test)**	
		Yes		8		105		
		No		29		617		
	**Liver disease**						**.43 (chi-square test)**	
		Yes		8		111		
		No		29		611		
	**Oxygenation index**						**.97 (chi-square test)**	
		Yes		2		28		
		No		35		694		
	**Respiratory index**						**.99 (chi-square test)**	
		Yes		0		10		
		No		37		712		
	**Heart failure**						**.84 (chi-square test)**	
		Yes		4		61		
		No		33		661		
	**Diabetes**						**.92 (chi-square test)**	
		Yes		12		218		
		No		25		504		
	**Coronary Sinus Pressure**						**.68 (chi-square test)**	
		Yes		0		18		
		No		37		704		
	**Dialysis**						**.85 (chi-square test)**	
		Yes		3		76		
		No		34		646		

^a^The digits outside the parentheses mean *P* value.

### Model Learning Results

As 5 cross-validation procedures were performed in this study, each of the 5 models was trained independently. Multimedia Appendix 1 shows the cost values over 1000 epochs for the training set and the validation set of each model. Although there were many points where the cost changed rapidly over the course of learning, the cost value decreased continuously over the epochs. From the first fold to the fifth fold, the cost value for the validation set was 0.217, 0.242, 0.271, 0.329, and 0.251 (Multimedia Appendix 1). As there is no clear criterion on when to stop training parameters during model training, we stopped model training with a heuristic approach based on the shape of the cost function. Specifically, the overall cost value hardly decreased when the epochs exceed 300. However, the cost values of both the training and validation sets suddenly increased after 500 epochs and settled after 700 epochs (Multimedia Appendix 1). To check if the cost instability was recaptured, we trained the model up to 1000 epochs and then stopped the learning.

### Time-Dependent Model Performance

**Figure 4:**
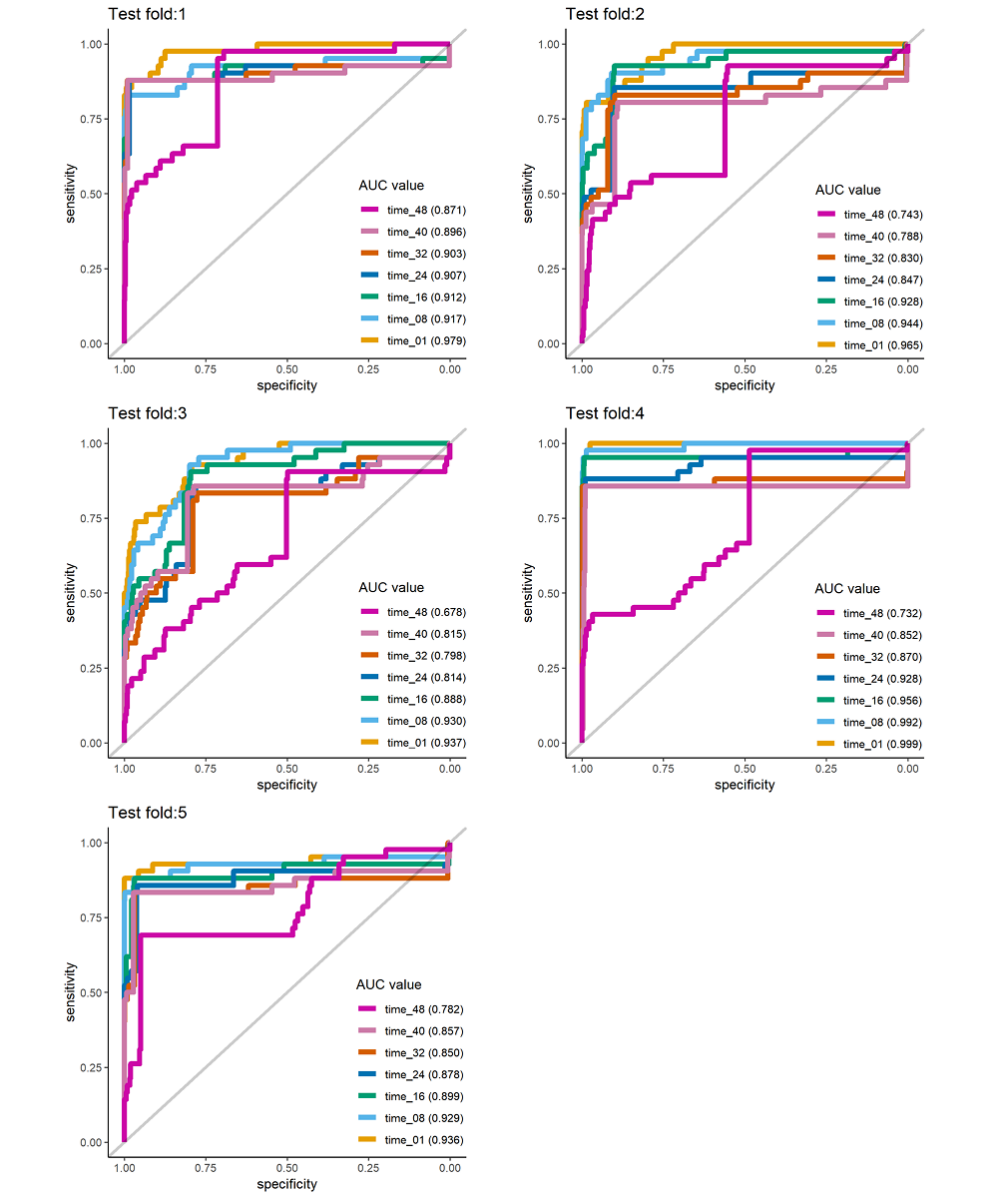
Results of time-dependent receiver operating characteristic analysis according to the fold change. AUC: area under the curve.

Overall, 5 time-dependent areas under the curve (TAUCs) were calculated using the aggregated set of 5 validation sets (Figure 4). In this study, these TAUCs were segmented according to 5 time points. The TTE equated to 1, 8, 16, 24, 32, 40, or 48 hours remaining to cardiac arrest in the cardiac arrest group and to be censored in the non–cardiac arrest group. The number of cardiac arrest cases according to the 5 folds was 41 (6.63%), 41 (6.63%), 42 (6.77%), 42 (6.77%), and 42 (6.77%). We show the performance of the 5 folds through the median for all time points (Multimedia Appendix 1). TAUCs for TTEs 1, 8, 16, 24, 32, 40, and 48 hours were calculated as 0.963, 0.942, 0.917, 0.875, 0.850, 0.842, and 0.761, respectively, indicating that model performance decreases as TTE increases. For all time points, the area under the curve of performance for the 5 folds is increased linearly (Multimedia Appendix 1). The average correlation coefficient between TAUC and time point in 5 folds was 0.910. Despite the smaller number of patients with cardiac arrest as compared with censored patients, the sensitivity ranged from 0.846 to 0.909. The specificity was generally high, ranging from 0.923 to 0.946, except for 48 hours, when there was a lack of prior information.

### Risk Score Comparison

**Figure 5:**
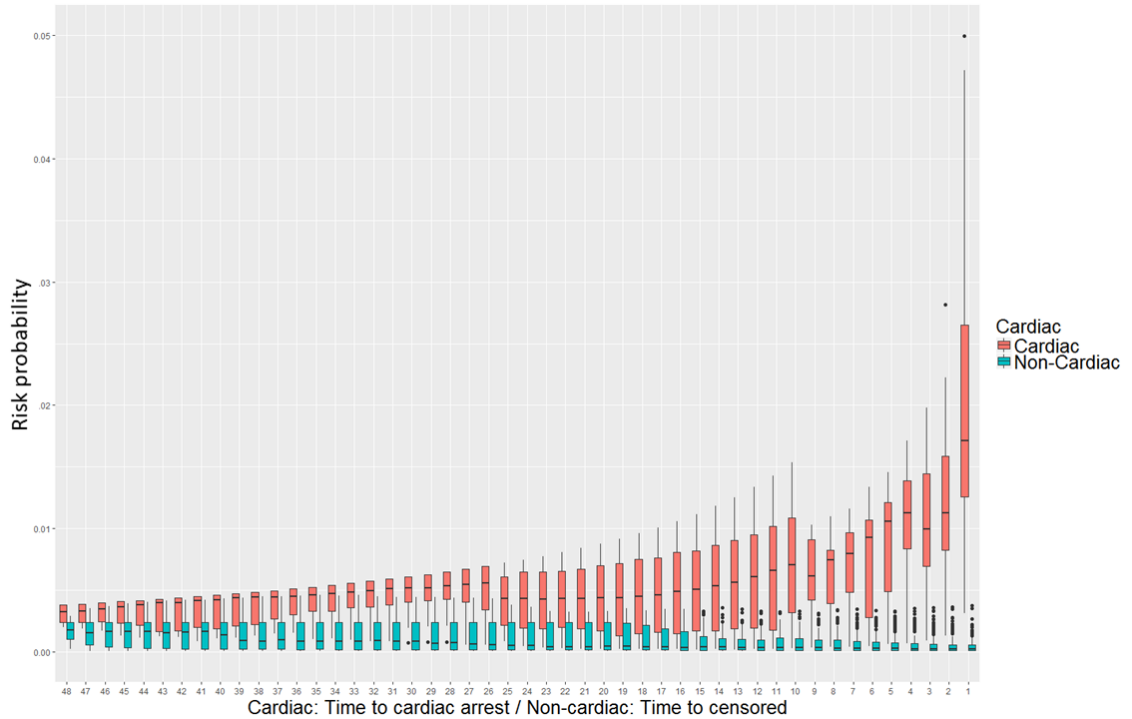
Risk probability comparison between cardiac arrest and the non–cardiac arrest groups. The x-axis represents the time point, and the y-axis represents the distribution of probability density values for cardiac arrest obtained for each patient corresponding to each time point.

Figure 5 shows how the risk probability in the cardiac arrest and the non–cardiac arrest groups changed over time. For the group with a cardiac arrest, the risk probability increases as the time for a cardiac arrest approaches. Conversely, the group without a cardiac arrest did not show an increase in the risk of cardiac arrest when the data were closer to the censored time. From 48 to 16 hours before cardiac arrest, the interquartile range (IQR) values overlap for the cardiac and noncardiac groups. However, the IQR of the risk probability of the 2 groups is separated from 15 hours ago. The median risk probability value from 15 hours ago also differs more than 10 times, and the difference continues to increase 1 hour before cardiac arrest.

### Predicted Cumulative Distribution Function at Time Point

**Figure 6:**
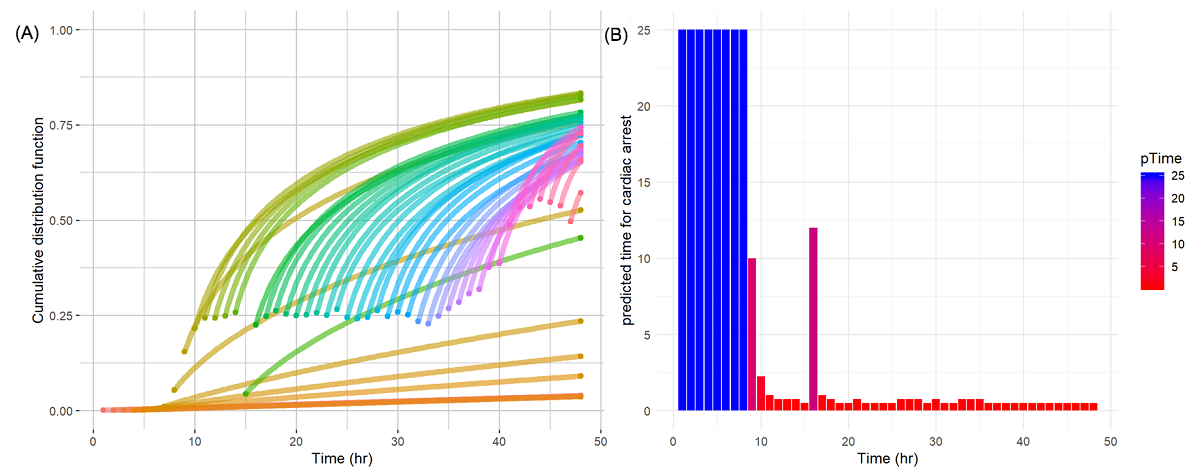
(A) Cumulative distribution function lines from the predicted time point to censoring time point for a patient with cardiac arrest at 48 time points; Each function line is color-coded. (B) Predicted hours remaining until a patient has cardiac arrest; the y-axis was limited to less than 25 hours for readability. pTime: predicted time.

An additional problem with arrest prediction is predicting when a cardiac arrest event will occur. A cumulative distribution function was derived through the shape and scale inferred by the model from each time point. Using the Weibull distribution parameter derived for the 48 time points, curves corresponding to cumulative distribution functions were drawn (A in Figure 6). Each line represents the probability of a cardiac arrest occurring from the start time point of the parameters until the remaining time. The closer the time to the cardiac arrest, the higher the beginning of the cumulative distribution function. This shows that even when the time point is far from cardiac arrest, a patient can be predicted to be high risk. Furthermore, the predicted time remaining until the patient has a cardiac arrest is presented (B in Figure 6). As the time approaches the cardiac arrest, the time remaining before the cardiac arrest occurs is estimated to be very small.

**Figure 7:**
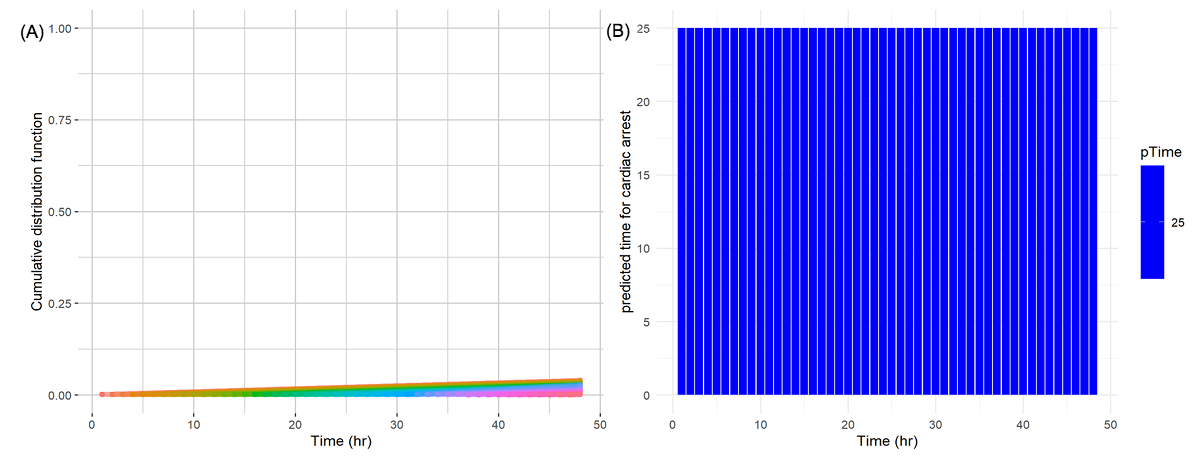
(A) Cumulative distribution function lines from the predicted time point to censoring time point for a patient without cardiac arrest at 48 time points; Each function line is color-coded. (B) Predicted hours remaining until a patient has cardiac arrest; the y-axis was limited to less than 25 hours for readability. pTime: predicted time.

Conversely, the distribution of the cumulative distribution function of a certain patient without cardiac arrest shows that, at all time points, the probability does not increase over time (A in Figure 7). Likewise, the time remaining until the patient has cardiac arrest is predicted to be very high (ie, more than 25 hours) over the entire time (B in Figure 7).

## Discussion

### Principal Findings

In this study, we developed the prediction model for cardiac arrest in critically ill patients through machine learning using electronic medical records. Besides vital sign, we used the underlying disease, laboratory data, medication, and organ failure as parameters to improve the accuracy of the prediction model. The TAUCs for TTE of 8, 16, and 24 hours were 0.942, 0.91, and 0.811, respectively, and the model performance decreased in accordance with increasing TTE.

#### Informative Outcomes

In previous studies related to cardiac arrest predictions, modeling techniques that predict whether an event occurs within a predefined predicted time window have often been implemented [4,9,12]. Although these approaches are efficient in terms of model implementation, these approaches have limitations because it is impossible to forecast whether cardiac arrest occurs or not outside a defined window or when it will occur. To overcome the limitations, we attempted to combine the Weibull distribution estimation technique with a Char-GRU. This modeling approach provides information about the cardiac arrest risk probability over the future time. Therefore, it is possible to provide an answer to how many hours are left until cardiac arrest occurs without a predefined time threshold that may limit the information. Thus, it is obviously much more informative to predict cardiac arrest in clinical practice.

#### Early Warning in Real Time

The early recognition of cardiac arrest and its prompt correction are critical to reducing the mortality of critically ill patients. To decide clinically who is unstable or who is going to deteriorate, many intensivists often scrutinize the vital signs of intensive care patients, such as blood pressure, heart rate, respiratory rate, and peripheral capillary oxygen saturation [21,22]. However, several studies have shown that these signs may not be effective in forecasting the risk early (ie, several hours before) [21,23]. This may be because of insufficient information on vital signs in predicting cardiac arrest in advance. However, considering that critical patient data are continuously generated in real time from numerous sources, including vital signs and information from organ support devices [12,13], the use of big data may provide models with sufficient information for the early prediction of cardiac arrest. Furthermore, the use of deep learning models, taking into account cumulative historical patterns of large clinical data, is expected to be very effective in predicting cardiac arrest in advance. In this regard, we implemented a deep learning–based model using a large dataset of 45 variables and found that the model could potentially be used for the early prediction of cardiac arrest.

#### Flexibility and Operational Reality

As cumulative and fluctuating effects of clinical variables over time can be reflected in deep learning algorithms, the use of long time series data to predict cardiac arrest is ideal. However, it is not appropriate to take no action until the patient has accumulated sufficient time series data. Waiting for sufficient time (ie, 48 hours) to accumulate patient time series data in clinical settings is undesirable for both patients and intensivists. Even if variables have not yet accumulated for a sufficient amount of time, a model should be available. In this situation, the Char-GRU structure allows the model to use the clinical variables. Specifically, the Char-GRU model can predict the risk of a patient’s cardiac arrest using clinical variables accumulated up to the present time (ie, 3 hours after entering the ICU) [17].

#### Estimation Efficiency

The early detection of disease onset is challenging in terms of the configuration of deep learning algorithm structures and data pipelines, as there is no reference for *early* time. Previous studies have been limited in predicting the onset of an illness just 1 time step ahead (a week or a month before) [24,25]. However, disease onset could be forecasted at various time points. For instance, the probability of 3 hours before, 2 hours before, and 1 hours before the onset of a disease may be predicted at the same time. In this setting, 3 dimensions—the number of observations, time steps, and disease indication (1=onset or 0=nononset)—should be considered in determining cardiac arrest. However, it is inefficient to predict cardiac arrest onsets over all time steps because it is unknown how many hours earlier the onsets should be predicted. However, when estimating the occurrence of a disease by estimating a Weibull distribution, the dimension of the outcome variables (ie, shape and scale parameters) is 2 (observation ID and Weibull distribution parameters—shape and scale). In other words, this setting estimates the time remaining until the onset of the disease in the form of a continuous variable so that the time dimension (ie, the cardiac arrest onset over time remaining) is removed from the outcome variable area. Therefore, this method is much more efficient because it can significantly reduce the need for various experiments.

### Limitations

This study has limitations that need to be addressed in further studies before applying Char-GRU with the Weibull distribution algorithm to clinics. In this study, rigorous validation was not performed while focusing on algorithm implementation using clinical data. As clinical data from only 1 medical institution were used, various additional validations are needed to generalize the results. To conduct rigorous validation, it is recommended to validate deep learning–based Weibull models using published data such as the modified early warning score [12].

Another limitation is the inability to fully control the reflection of certain effects in the collected data, which may affect the model results. For instance, data from a treated patient who is perceived to be in a very dangerous condition may cause a bias against the time series characteristics in the high-risk group [26]. In other words, the patient’s data reflecting the time series characteristics of the non–cardiac arrest group would ultimately reflect the time series characteristics of the cardiac arrest group if the patient had not been treated [26,27]. In the previous studies, such data were just removed or corrected based on statistical methods [26,28]. Therefore, further research that validates this algorithm requires in-depth consideration of data selection and preprocessing.

### Conclusions

The cardiac arrest survival rate in hospitals is about 24%, and even after survival, patients suffer from fatal problems such as brain damage [29,30]. However, because of the difficulty in forecasting cardiac arrest in advance, adequate prior interventions were rarely provided. We hope that the early prediction of cardiac arrest is linked to early intervention for the prevention of cardiac arrest. For that purpose, further research is essential to discuss how to operate deep learning models linked with a database and what forms of model outcomes should be provided to medical providers in practice.

## Appendix

Multimedia Appendix 1 Supplementary figures and tables.

## Abbreviations

Char-GRU: character-level gated recurrent unit
GRU: gated recurrent unit
ICU: intensive care unit
IHCA: in-hospital cardiac arrest
IQR: interquartile range
MICU: medical intensive care unit
ROC: receiver operating characteristic
TAUC: time-dependent area under the curve
TTE: time to event
