# Supplementary material for: Development of a Real-Time Risk Prediction Model for In-Hospital Cardiac Arrest in Critically Ill Patients Using Deep Learning: Retrospective Study
Source: JMIR Med Inform. 2020 Mar 18;8(3):e16349. doi: 10.2196/16349 (PMC7113801; doi:10.2196/16349)
Supplement: Multimedia Appendix 1 [file medinform_v8i3e16349_app1.pdf]

Multimedia Appendix

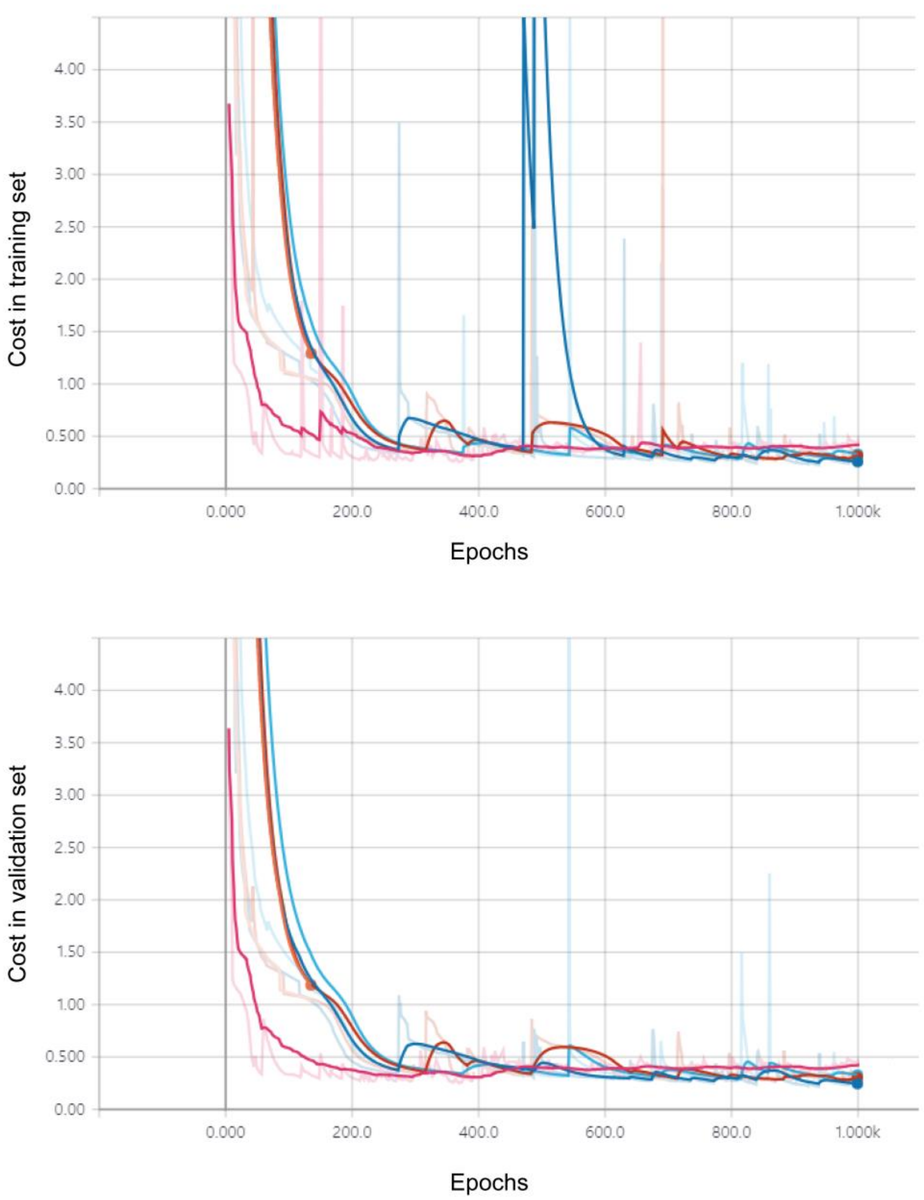

Figure A1. Cost values over epochs of five-fold validation sets. The Y-axis is the cost value in the training, which is a negative log of the likelihood derived from the shape and scale parameters of the Weibull model.

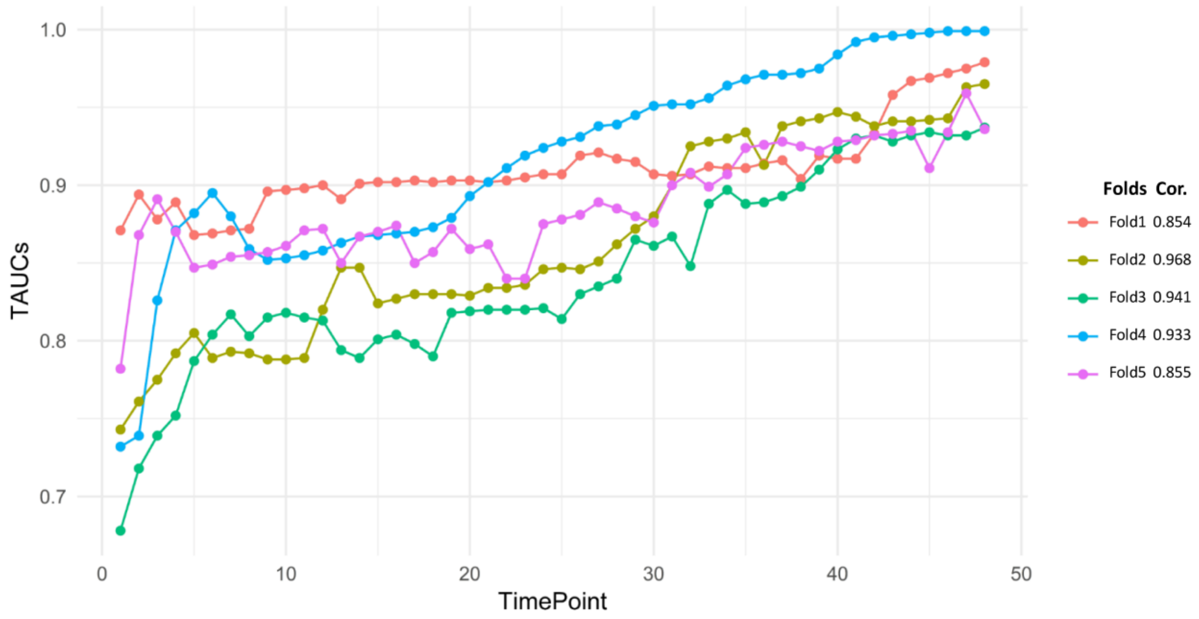

Figure A2. Pearson correlation analysis of the TAUCs and time points. For all time points, the AUC value of the performance for the 5-cross validation set is plotted as a line chart. Pearson correlation was performed for time points and AUCs.

Table A1. List of categorized clinical data by patient demographics, diagnosis, medication, vital signs, medication, and inputs and outputs

Categories		Variables	N	Obs.
Demographics	Time fixed	Age, gender	1,318	1,318 (Time fixed)
	Time varying	Weight	1,309	268,947
Diagnosis (Time fixed)		d_HM, d_LD, d_OI, d_RI, d_Meta, d_HF, d_Diab, CSP, Dialysis	1,324	1,324 (Time fixed)
Lab		BE, BiC, CO2, Cr, FiO2, Lac, pO2, PLT, WBC, K, Hct, Na, EANC, Hb, O2, ApH	1,212	221,765
Sofa		ResSOFA, CoagSOFA, LivSOFA, CardioSOFA, NervSOFA, RenalSOFA	1,235	203,624
Vitals		Rr, hr, sbp, dbp, temp, map, si	1,308	253,008
Medication		Amiodarone, Epinephrine, Norepinephrine, Vasopressin	696 [981]	981 (Time fixed)

Table A2. Cost in training and validation sets of models with the lowest cost among 1,000 epochs from Fold 1 to Fold 5.

**Fold number**	**Cost in Training**	**Cost in Validation**
Fold-1	0.2261	0.2172
Fold-2	0.2525	0.2419
Fold-3	0.2694	0.2706
Fold-4	0.3219	0.3288
Fold-5	0.2540	0.2513

Table A3: Median value of the performance of the 5-cross validation set for all time points (48 h).

Time Point	AUC	Accuracy	Sensitivity	Specificity	PVP	PVN
Time_48	0.743	0.578	0.927	0.553	0.009	0.872
Time_47	0.761	0.735	0.881	0.726	0.012	0.819
Time_46	0.826	0.788	0.881	0.785	0.011	0.785
Time_45	0.870	0.902	0.857	0.905	0.011	0.604
Time_44	0.847	0.947	0.854	0.952	0.010	0.431
Time_43	0.849	0.955	0.854	0.962	0.010	0.375
Time_42	0.854	0.961	0.854	0.971	0.010	0.327
Time_41	0.855	0.963	0.854	0.972	0.012	0.314
Time_40	0.852	0.963	0.857	0.972	0.012	0.314
Time_39	0.853	0.961	0.857	0.971	0.012	0.327
Time_38	0.855	0.961	0.857	0.969	0.011	0.333
Time_37	0.858	0.956	0.857	0.964	0.011	0.368
Time_36	0.850	0.955	0.854	0.964	0.011	0.375
Time_35	0.867	0.960	0.857	0.967	0.011	0.345
Time_34	0.868	0.956	0.857	0.964	0.011	0.368
Time_33	0.869	0.963	0.857	0.971	0.011	0.321
Time_32	0.850	0.960	0.833	0.969	0.012	0.340
Time_31	0.857	0.958	0.833	0.967	0.012	0.352
Time_30	0.872	0.953	0.857	0.960	0.011	0.390
Time_29	0.859	0.955	0.833	0.964	0.012	0.375
Time_28	0.862	0.955	0.857	0.964	0.012	0.375
Time_27	0.840	0.948	0.857	0.958	0.013	0.414
Time_26	0.840	0.948	0.857	0.958	0.013	0.414
Time_25	0.875	0.952	0.857	0.958	0.011	0.400
Time_24	0.878	0.953	0.857	0.960	0.011	0.390
Time_23	0.881	0.963	0.878	0.971	0.011	0.321
Time_22	0.889	0.966	0.878	0.974	0.011	0.294
Time_21	0.885	0.963	0.878	0.972	0.011	0.314
Time_20	0.880	0.963	0.878	0.972	0.009	0.314
Time_19	0.880	0.961	0.878	0.972	0.010	0.320
Time_18	0.900	0.958	0.878	0.965	0.010	0.357
Time_17	0.908	0.956	0.881	0.962	0.009	0.373
Time_16	0.912	0.963	0.905	0.969	0.009	0.327
Time_15	0.911	0.961	0.927	0.967	0.007	0.339

Time_14	0.924	0.956	0.905	0.962	0.009	0.373
Time_13	0.914	0.956	0.902	0.962	0.008	0.373
Time_12	0.928	0.961	0.927	0.967	0.006	0.339
Time_11	0.925	0.960	0.881	0.967	0.011	0.345
Time_10	0.922	0.965	0.927	0.972	0.006	0.308
Time_09	0.928	0.965	0.905	0.971	0.009	0.315
Time_08	0.930	0.965	0.902	0.971	0.008	0.315
Time_07	0.933	0.976	0.905	0.981	0.007	0.224
Time_06	0.941	0.971	0.905	0.976	0.007	0.269
Time_05	0.941	0.974	0.878	0.979	0.009	0.240
Time_04	0.942	0.982	0.854	0.984	0.010	0.180
Time_03	0.943	0.987	0.854	0.995	0.010	0.083
Time_02	0.963	0.973	0.854	0.979	0.010	0.267
Time_01	0.965	0.974	0.881	0.977	0.009	0.250
